# De Novo Malignancies after Liver Transplantation: A Multicenter Cohort Study of Incidence, Timing, and Outcomes*

**DOI:** 10.5152/tjg.2026.25748

**Published:** 2026-02-20

**Authors:** Nilay Danış, Hüseyin Döngelli, Tarkan Ünek, Aylin Bacakoğlu, İlker Turan, Abdullah Zeki Karasu, Fulya Günşar, Elvan Işık, Muhsin Murat Harputluoglu, Kutay Sağlam, Sezai Yılmaz, Bilger Çavuş, Sabahattin Kaymakoglu, Haydar Adanır, Dinç Dinçer, Derya Arı, Meral Akdogan Kayhan, Dilara Turan Gokce, Gökhan Kabacam, Sedat Karademir, Murat Kıyıcı, Selcan Cesur, Hale Gokcan, Zeynep Melekoglu Ellik, Ramazan İdilman, Murat Dayangaç, Yasemin Balaban, Ahmet Bülent Doğrul, Hilmi Anıl Dinçer, Gupse Adalı, Feyza Dilber, Murat Taner Gulsen, Orhan Sezgin, Zekiye Nur Harput, Murat Akyıldız, Kenan Moral, Tufan Egeli, Cihan Ağalar, Mücahit Özbilgin, Mesut Akarsu

**Affiliations:** 1 Liver Transplantation Center, Dokuz Eylül University Hospital, İzmir, Türkiye; 2 Liver Transplantation Center, Ege University Hospital, İzmir, Türkiye; 3 Liver Transplantation Center, İnonu University Hospital, Malatya, Türkiye; 4 Liver Transplantation Center, İstanbul University Hospital, İstanbul, Türkiye; 5 Liver Transplantation Center, Akdeniz University Hospital, Antalya, Türkiye; 6 Liver Transplantation Center, Ankara Bilkent City Hospital, Ankara, Türkiye; 7 Liver Transplantation Center, Güven Hospital, Ankara, Türkiye; 8 Liver Transplantation Center, Bursa Uludağ University Hospital, Bursa, Türkiye; 9 Liver Transplantation Center, Ankara University Hospital, Ankara, Türkiye; 10 Liver Transplantation Center, İstanbul Medipol University Hospital, İstanbul, Türkiye; 11 Liver Transplantation Center, Hacettepe University Hospital, Ankara, Türkiye; 12 Liver Transplantation Center, Ümraniye Training and Research Hospital, İstanbul, Türkiye; 13 Liver Transplantation Center, Marmara University Hospital, İstanbul, Türkiye; 14 Liver Transplantation Center, Gaziantep University Hospital, Gaziantep, Türkiye; 15 Liver Transplantation Center, Mersin University Hospital, Mersin, Türkiye; 16 Liver Transplantation Center, Koç University Hospital, İstanbul, Türkiye; 17 Liver Transplantation Center, Ankara Gazi University Hospital, Ankara, Türkiye

**Keywords:** Immunosuppression, liver transplantation, neoplasms, postoperative care, prognosis

## Abstract

**Background/Aims:**

De novo malignancies are an important long-term complication following liver transplantation (LT), driven by chronic immunosuppression and extended post-transplant survival. Understanding patterns by tumor type, timing, and patient characteristics is essential for optimized surveillance. This study aimed to evaluate the clinical characteristics and temporal trends of de novo neoplasms in LT recipients.

**Materials and Methods:**

A total of 6943 adult LT recipients from 16 Turkish centers were evaluated between 1999 and 2023). Two hundred eight patients with 220 histologically confirmed de novo malignancies (excluding hepatocellular carcinoma) were included. Patients who died within 30 days or developed cancer within 90 days post-LT were excluded. Incidence and mortality rates were assessed across post-transplant intervals.

**Results:**

Non-skin solid (53.2%), dermatologic (28.6%), and hematologic (18.2%) cancers were predominant. Most malignancies (76.8%) occurred within 10 years post-LT. Early-onset (<10 years) malignancies were more common in living donor recipients (62.7%, *P* = .001) and associated with higher mortality (39.6% vs. 11.8%, *P* < .001). Recipients ≤60 years exhibited a higher incidence of hematologic cancers (23.9% vs. 12.6%, *P* = .03) and increased mortality (39.4% vs. 27.0%, *P* = .05). In subgroup analysis, tacrolimus use was strongly associated with cutaneous squamous cell carcinoma (*P* = .004). Peak incidence occurred 2-10 years post-LT (≈13-14 per 1000 recipients), with non-skin solid tumors being the most frequent.

**Conclusion:**

De novo malignancies remain a significant long-term risk after LT, with outcomes influenced by tumor type, age, immunosuppression, and timing of onset. Risk-adapted surveillance and tailored oncologic management, particularly dermatologic screening in tacrolimus-treated patients and enhanced surveillance during the 2-10 year post-transplant period are recommended to improve long-term survival.

**Main Points**

- Early-onset (<10 years) post-transplant malignancies are associated with significantly higher mortality, especially among younger recipients and those receiving living-donor grafts.
- Age at malignancy diagnosis strongly influences cancer patterns and outcomes: younger recipients show increased rates of hematologic cancers and worse survival, while older recipients more frequently develop solid tumors and undergo surgical management.
- Tacrolimus-based immunosuppression is significantly associated with cutaneous squamous cell carcinoma, underscoring the importance of dermatologic surveillance in this subgroup.
- Peak cancer incidence occurs 2-10 years post-transplant, highlighting the need for risk-adapted, time-specific surveillance and tailored oncologic strategies to improve long-term outcomes.

## Introduction

Liver transplantation (LT) is a life-saving treatment modality for various conditions, including liver cirrhosis, fulminant hepatic failure, and hepatocellular carcinoma (HCC).^1^ Although significant advancements have been made over the past 2 decades in surgical procedures, immunosuppression protocols, and perioperative and postoperative care, short- and long-term complications remain considerable challenges.^2^ Moreover, with progressive improvement in patient survival, long-term complications have become increasingly prominent.^3^

In the first year following LT, approximately 1 in 10 patients die.^4^ In the initial weeks, hemorrhage and mechanical surgical complications are the leading causes of death.^4^ In a population-based study that compared the survival of liver transplant recipients who lived beyond the first post-transplant year with that of the general population, transplant-specific mortality accounted for 16% of all deaths.^5^ Malignancy was identified as the most frequent cause of death, followed by infections as the second most common, and cardiovascular diseases as the third.^5^ In another study, cancer was identified as the leading cause of death among liver transplant recipients, followed by infections, graft failure, and cardiovascular disease.^6^

Posttransplant malignancies are now recognized as one of the leading causes of late mortality in liver transplant recipients.^7^ Compared to the general population, transplant recipients face a markedly increased risk of developing certain cancers, which is attributed primarily to chronic immunosuppression, viral infections, and pre-existing risk factors.^7^ While essential for graft survival, immunosuppressive therapy compromises immune surveillance and facilitates oncogenesis.^8^ While several studies have implicated specific aspects of immunosuppressive treatment in increasing the risk of de novo malignancies, others have found no significant correlation.^9^^-^^11^

Among post-transplant de novo malignancies, the most frequently observed types, in descending order of prevalence, are hematologic cancers, non-melanoma skin cancers, head and neck cancers, lung cancer, prostate cancer, and colorectal cancer.^9^^,^^10^

This multicenter retrospective study aimed to evaluate the incidence, types, and timing of de novo malignancies among liver transplant recipients in the posttransplant period, which may guide future surveillance and management strategies.

## Materials and Methods

This multicenter, retrospective cohort study included adult liver transplant recipients diagnosed with histopathologically confirmed de novo malignancies, excluding HCC. The study population comprised 6943 patients who underwent LT between 1999 and 2023 at 16 transplant centers across 8 cities in Türkiye. Two hundred and eight recipients developed post-transplant de novo malignancies (n = 220) and were included in the final analysis.

The study was conducted and reported in accordance with the STROBE (Strengthening the Reporting of Observational Studies in Epidemiology) guidelines.

### Data Collection

Comprehensive clinical, demographic, and transplant-related data were retrospectively extracted from the institutional databases. The variables collected included recipient age, sex, body mass index (BMI), blood type, Model for End-stage Liver Disease (MELD) score, donor type (living or deceased), presence of HCC at the time of transplantation, recipient and donor history of malignancy (for living donors), family history of cancer, and comorbidities. Post-transplant parameters encompass immunosuppressive regimens, the number of biopsy-proven acute rejection episodes, age at diagnosis of de novo malignancy, time from transplantation to malignancy diagnosis, and treatment modalities. Complete longitudinal follow-up data for the entire transplant cohort (N = 6943) were not uniformly available across centers; therefore, median follow-up duration could only be reported for patients who developed malignancy.

### Inclusion and Exclusion Criteria

**Figure 1.**
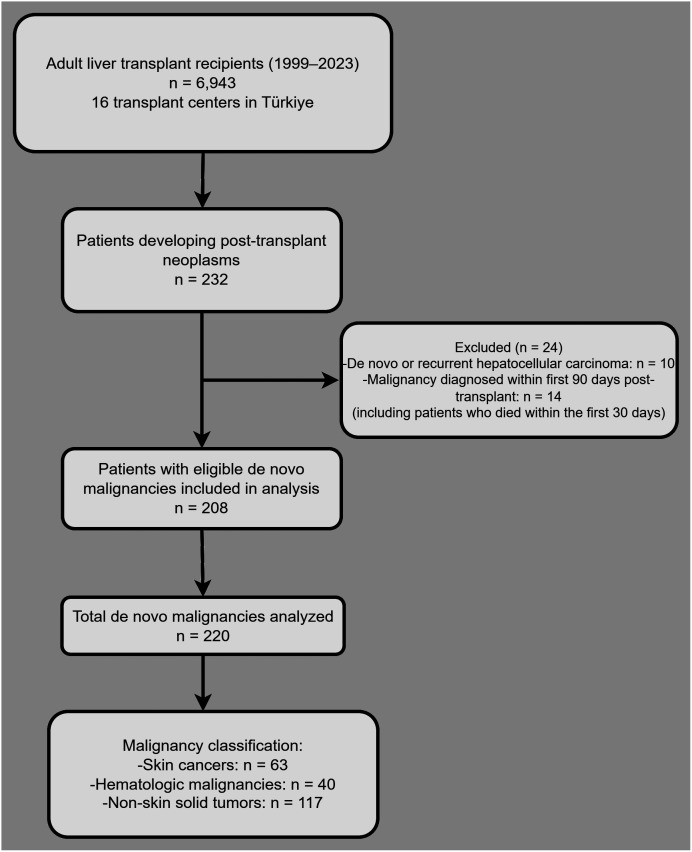
Flowchart showing patient selection.

Eligible patients were those who underwent LT and subsequently developed de novo malignancy confirmed by histopathological examination. Patients with recurrent or de novo HCC were excluded to focus exclusively on non-hepatic de novo malignancies. Patients who died within 30 days and those who had a diagnosis of cancer within 90 days after LT were excluded (Figure 1). In cases in which more than 1 malignancy occurred in a single recipient, each tumor was recorded and analyzed as a separate event with individual diagnostic and pathological characteristics.

### Classification and Temporal Analysis of Malignancies

All de novo malignancies identified in the transplant cohort (N = 6943) were classified into 4 major histological categories: skin cancers, hematologic malignancies, non-skin solid tumors, and connective tissue neoplasms. The temporal distribution was analyzed across 3 post-transplant intervals: 0-2 years, 2-10 years, and >10 years. Incidence rates were calculated as the number of malignancies per 1000 transplant recipients within each predefined post-transplant interval. Person-time–based incidence rates were not calculated due to heterogeneity in follow-up duration across centers.

Patient-level and neoplasm-level analyses were reported separately to avoid methodological overlap, particularly in recipients who developed multiple de novo malignancies.

The cumulative incidence was determined by summing all the cases within each histological group. Mortality was assessed at each time interval to evaluate longitudinal trends. Tumor-based analyses were performed for malignancy characteristics and incidence, whereas mortality and demographic analyses were conducted at the patient level.

### Ethical Considerations

The study was designed as a multicenter retrospective cohort study and conducted across the following tertiary-care institutions in Türkiye: Dokuz Eylül University Hospital, Ege University Hospital, İnönü University Hospital, İstanbul University Hospital, Akdeniz University Hospital, Ankara Bilkent City Hospital, Ankara Güven Hospital, Bursa Uludağ University Hospital, Ankara University Hospital, İstanbul Medipol University Hospital, Hacettepe University Hospital, Ümraniye Training and Research Hospital, Marmara University Hospital, Gaziantep University Hospital, Mersin University Hospital, Koç University Hospital, and Ankara Gazi University Hospital. The study protocol was approved by the institutional review board of Dokuz Eylül University (Approval number: 2025/28-25, August 20, 2025). The ethics committee waived the requirement for informed consent owing to the retrospective nature of the study. The study was conducted in accordance with the principles of the Declaration of Helsinki, 2018 Declaration of Istanbul, and adhered to the STROBE (Strengthening the Reporting of Observational Studies in Epidemiology) guidelines for observational cohort studies.

### Statistics

Continuous variables are presented as mean ± standard deviation (SD) or median with interquartile range (IQR), depending on the distribution. Categorical variables are reported as counts and percentages. Group comparisons between living and deceased donor recipients were conducted using independent sample *t*-tests or Mann–Whitney *U*-tests for continuous variables and chi-square or Fisher’s exact tests for categorical variables, as appropriate.^1^^,^^4^ Overall survival after de novo malignancy diagnosis was analyzed using the Kaplan–Meier method. Survival curves were compared using the log-rank test for malignancy type (skin vs. non-skin) and timing of onset (0-10 years vs. >10 years post-transplant). A 2-sided *P *value <.05 was considered statistically significant. All statistical analyses were performed using SPSS version 26 (IBM SPSS Corp.; Armonk, NY, USA).

## Results

### Patient Characteristics and Comparison by Donor Type

Among 6943 liver transplant recipients, 208 developed post-transplant de novo malignancies and were included in the study cohort. Of these, 91 (43.8%) received grafts from deceased donors and 117 (56.2%) received grafts from living donors. The baseline demographic and clinical characteristics, including age at transplantation (*P *= .357), sex (*P* = .756), BMI (*P* = .175), and MELD score at listing (*P* = .297), were comparable between the 2 donor groups. There were no significant differences in lifestyle factors (smoking and alcohol use), underlying liver disease etiology, or pre-transplant HCC presence (*P* = .383). Immunosuppressive regimens and comorbidities were similar across groups (Table 1).

Dermatologic (23.1%) and hematologic (20.2%) cancers were the most common neoplasm types, with comparable distributions between the donor types. Multiple de novo malignancies occurred in 7 patients (3.4%) (Table 1). The overall mortality rate following malignancy diagnosis was 35.1%, with no significant difference between recipients of deceased and living donor grafts (*P* = .784).

### Early Vs. Late-Onset Malignancies

In total, 220 de novo malignancies were diagnosed in 208 patients. Of these, 169 (76.8%) occurred within the first 10 years post-transplantation (early onset group), whereas 51 (23.2%) were diagnosed beyond 10 years (late-onset group) (Table 2). Patients with late-onset malignancies were significantly older at the time of cancer diagnosis (61.4 ± 10.5 vs. 57.0 ± 12.6 years; *P* = .026) and more frequently received grafts from deceased donors (*P* = .001). Other baseline characteristics, including sex, BMI, primary liver disease, comorbidities, and immunosuppressive protocols, were not significantly different.

Multiple malignancies were more frequent in the late-onset group (15.7% vs. 5.9%, *P* = .041). Mortality was significantly higher in the early onset group (39.6% vs. 11.8%, *P* < .001) despite similar therapeutic strategies (Table 2).

### Malignancy Characteristics by Age at Diagnosis

When stratified by age at malignancy diagnosis (≤60 vs. >60 years), several differences emerged. Female recipients were more common among younger patients (43.1% vs. 27.0%, *P* = .012), whereas pretransplant HCC was more prevalent in older individuals (37.8% vs. 16.5%, *P* < .001). Tacrolimus was more commonly used in the younger group (88.1% vs. 77.5%, *P* = .038), whereas older recipients more frequently received mycophenolate mofetil (56.8% vs. 36.7%, *P* = .003) and everolimus (41.4% vs. 22.9%, *P* = .003) (Table 3).

Hematologic malignancies were significantly more frequent in the younger cohort (23.9% vs. 12.6%, *P* = .031), whereas prostate cancer was more common among older patients (9.0% vs. 0.9%, *P* = .010). Surgical treatment was performed more frequently in the older group (66.7% vs. 46.8%, *P* = .003). A trend toward higher mortality was observed in the younger group (39.4% vs. 27.0%, *P* = .050), although this was not statistically significant. The time from transplantation to neoplasm diagnosis and the post-diagnosis follow-up duration were similar between the 2 groups (Table 3).

### Comparison of Skin Vs. Non-Skin Malignancies

Among the 220 malignancies, 63 (28.6%) were classified as skin cancers and 157 (71.4%) were classified as non-skin cancers. Malignant melanoma developed in 2 patients only. No significant differences were observed between the groups with respect to age, sex, BMI, blood group, or donor type (Table 4). However, the median post-diagnosis observation time was significantly longer for skin cancer patients (45 vs. 22 months, *P* = .001). Multiple neoplasms were more common in patients with skin cancer than in those without (19.0% vs. 3.8%, *P* < .001). Management strategies differed notably; surgical intervention was the primary treatment for skin cancers (95.2%), whereas chemotherapy and combination therapies were more often used for non-skin malignancies (both *P* < .001). Additionally, in the subgroup analysis of patients who developed cutaneous squamous cell carcinoma, 39 of 40 patients were receiving tacrolimus (*P *= .004). Among 7 patients who developed pancreatic cancer, 6 were on mycophenolate mofetil therapy (*P* = .050). The mortality rate was significantly lower in patients with skin neoplasms (7.9% vs. 43.3%, *P* < .001) (Table 4).

### Temporal Trends in Incidence and Mortality

**Figure 2.**
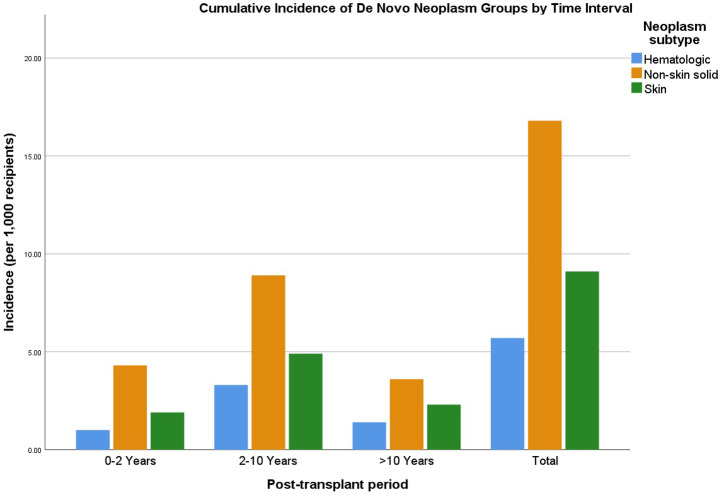
Incidence of de novo neoplasms according to post-transplant interval.

The cumulative incidence of post-transplant neoplasms varied across different time intervals. During the first 0-2 years (excluding posttransplant 3 months) post-LT, 50 malignancies were identified, corresponding to an incidence of 7.2 per 1000 recipients, with skin (n = 13; 1.9/1000), hematologic (n = 7; 1.0/1000), and non-skin solid (n = 30; 4.3/1000). Between 2 and 10 years post-transplant, malignancy incidence peaked, with 119 cases (17.1/1000). Non-skin solid tumors were most frequent (n = 62; 8.9/1000), followed by skin (n = 34; 4.9/1000), and hematologic (n = 23; 3.3/1000). Beyond 10 years, 51 malignancies (7.3/1000) were recorded, including 25 non-skin solid (3.6/1000), 16 skin (2.3/1000), and 10 hematologic tumors (1.4/1000). Mortality in this late period was lowest, with 6 deaths (0.9/1000). Overall, across the entire cohort (N = 6943), 220 de novo malignancies were identified, representing a cumulative incidence of 31.6 per 1000 recipients, with 73 deaths (1.1/1000) (Figure 2).

**Figure 3.**
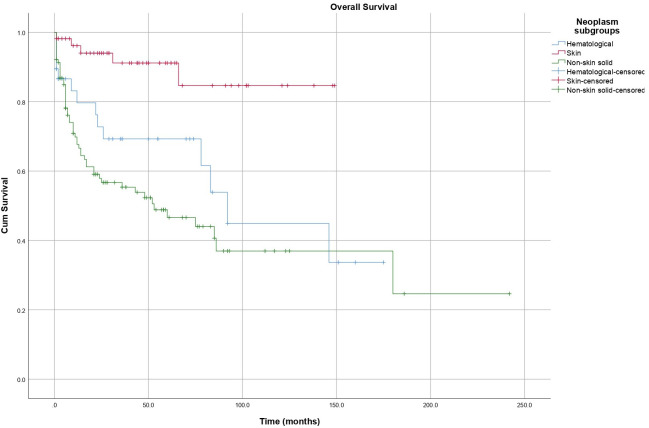
Kaplan–Meier curves showing overall survival by malignancy subtype (*P* < .001).

Overall survival differed significantly according to malignancy subtype, with the most favorable outcomes observed in dermatological malignancies, followed by hematological malignancies, whereas non-skin solid tumors were associated with the poorest survival (log-rank *P* < .001) (Figure 3).

**Figure 4.**
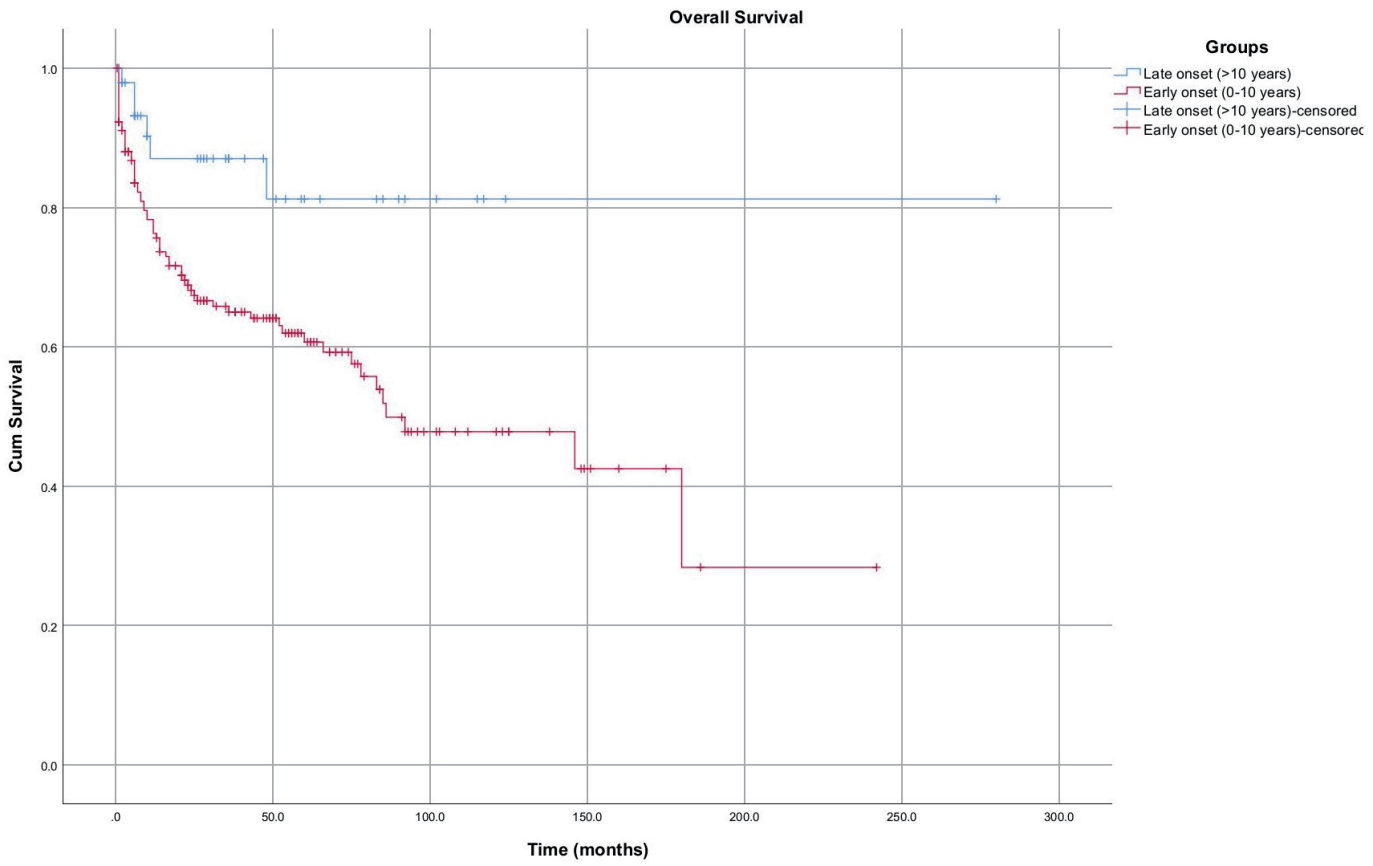
Kaplan–Meier curves showing overall survival by early vs. late onset neoplasm (*P *= .006).

Kaplan–Meier analysis demonstrated significantly poorer overall survival in patients with early-onset de novo malignancies (0-10 years post-transplant) compared with those with late-onset malignancies (>10 years) (log-rank *P* = .006) (Figure 4).

## Discussion

This multicenter retrospective study provides a comprehensive analysis of post-transplant de novo malignancies among liver transplant recipients in Türkiye, highlighting critical differences in clinical presentation and outcomes based on tumor type, timing of onset, and patient age. Most malignancies occur within the first 10 years post-transplant, with non-skin solid, dermatologic, and hematologic cancers comprising the most common subtypes. Early-onset malignancies were associated with significantly higher mortality rates, despite similar treatment strategies. Age at diagnosis was a key determinant of the cancer profile and management; younger recipients exhibited a higher incidence of hematologic malignancies and greater mortality, whereas older patients more commonly developed solid cancer and underwent surgical treatment. Patients with skin neoplasms have markedly better survival rates. They were predominantly treated surgically, in contrast to those with non-skin tumors who required more intensive therapies and had substantially worse outcomes.

The demographic and clinical characteristics of the cohort were largely consistent with those of previously published studies on post-transplant malignancies.^8^^-^^14^ The mean age at transplantation (61.6 years) and male predominance (64.9%) aligned with the global liver transplant trends.^1^ Living donor transplantation was slightly more common (56.2%), consistent with national practices in Türkiye, where living-donor LT is frequently performed.^15^^,^^16^ Hepatitis B virus-related cirrhosis remained the most common etiology, in line with regional epidemiological patterns.^17^ These findings suggest that the patient population is broadly representative and support the generalizability of the study results within similar settings.

Among 6943 liver transplant recipients, 208 developed de novo malignancies during the follow-up period, corresponding to a cumulative incidence of 29.8 cases per 1000 recipients. Most de novo malignancies in the cohort occurred within the first 10 years post-transplant, consistent with prior studies indicating an increased cancer risk during early intensive immunosuppression.^10^^-^^14^ Early-onset cancers were associated with significantly higher mortality, consistent with previous studies, despite similar treatment strategies, suggesting a more aggressive tumor behavior or host vulnerability.^4^^,^^5^ Interestingly, the distribution of cancer types was comparable between the early and late periods, but multiple malignancies were more frequent after 10 years, which reflects cumulative risk over time.

The analysis revealed that skin neoplasms in liver transplant recipients differ markedly from non-skin malignancies in terms of clinical course and prognosis. Patients with cutaneous cancers had significantly better survival rates and were predominantly managed with surgery, consistent with the localized nature and earlier detection of these tumors. Moreover, most skin cancers were non-melanoma types, which are known to have a limited impact on overall mortality.^9^ These findings align with those of previous studies emphasizing favorable outcomes for post-transplant skin cancers owing to their indolent behavior and accessibility for screening ^9^^,^^18^

Notably, a greater proportion of patients with skin neoplasms were on tacrolimus-based immunosuppression, supporting the evidence that calcineurin inhibitors are associated with increased skin cancer risk.^19^^,^^20^ While tacrolimus is essential for graft protection, it impairs immune surveillance and DNA repair, contributing to carcinogenesis.^14^ Although dose-dependent effects of tacrolimus on skin cancer risk have been described in prior studies, detailed dosage and trough level data were not available in the cohort. Conversely, mTOR inhibitors have been associated with a reduced risk of cutaneous malignancies and may represent a protective strategy in high-risk patients. ^21^^,^^22^

The study design did not allow for the evaluation of risk factors; however, several studies have identified potential risk factors for various types of de novo cancers in liver transplant recipients, including male sex, older age, multiorgan transplantation, and alcoholic liver disease.^13^^,^^14^^,^^23^ Conversely, the impact of immunosuppressive regimens on the development of de novo malignancies remains a subject of ongoing debate.^14^^,^^23^^,^^24^

In contrast, patients with non-skin cancers had poorer outcomes and were more likely to require systemic therapy. Their often-late presentation underscores the need for improved surveillance beyond dermatological evaluation. These observed patterns underscore the need for individualized surveillance strategies in liver transplant recipients. Customized follow-up should align with each patient’s risk profile, taking into account factors such as tumor histology, age at malignancy onset, and timing post-transplant, and optimizing the immunosuppressive regimen by balancing oncologic risk with graft protection.^24^

The higher rate of early-onset malignancies among living donor recipients in the study may reflect inherent differences in recipient selection, including younger age at transplantation and longer post-transplant survival.

The study has several limitations. First, owing to its retrospective design, causal relationships between clinical variables and outcomes cannot be definitively established. However, the study provides valuable real-world data from a large cohort of liver transplant recipients, with a notably higher proportion of living donor transplants compared to previous studies, offering highly relevant insights into this population in clinical practice. Second, although the study involved 15 liver transplant centers across Türkiye, not all transplant centers were included. Differences in cancer screening practices between centers might have introduced a detection bias, potentially underestimating the true incidence of specific malignancy subtypes. As post-transplant follow-up protocols and management strategies may vary among centers, this could introduce heterogeneity in the data. Nonetheless, the multicenter design enhances the external validity of the findings. Third, complete baseline and follow-up data were not available for the entire cohort of 6943 liver transplant recipients. This limits the ability to perform comprehensive risk factor analysis or comparative evaluations between patients with and without de novo malignancies. Fourth, data on therapeutic approaches for de novo malignancies were retrospectively collected from hospital databases, which may not have captured all aspects of clinical decision-making or treatment modifications over time. Fifth, only all-cause mortality was analyzed, as cause-specific mortality (e.g., cancer-related death) could not be reliably determined from the available records because of the retrospective nature of the study. Another limitation is that immunosuppressive regimens were recorded based on agent type only, without detailed information on dosages, duration, or changes over time, which may have influenced the risk of cancer and outcomes. Finally, multiple de novo malignancies in the same patient were analyzed as separate events. Analyzing multiple malignancies as separate events may overrepresent patients with recurrent or multiple tumors and should be considered when interpreting tumor-based incidence estimates.

In conclusion, post-transplant de novo malignancies remain a significant long-term complication of LT, with distinct patterns according to age, tumor type, and timing of onset. Non-skin solid and hematologic malignancies were more common in younger recipients and were associated with worse survival, whereas skin neoplasms demonstrated favorable outcomes with predominantly surgical management. The elevated mortality in early-onset cases and age-related variation in immunosuppressive regimens highlight the need for risk-adapted surveillance and tailored oncologic strategies to improve outcomes in this growing patient population.

## Figures and Tables

**Table 1.** Patient-Based Clinical and Transplant-Related Characteristics of Liver Transplant Recipients with De Novo Malignancies, Stratified by Donor Type (N = 208)

	**Cadaver Donor (n = 91)**	**Living Donor (n = 117)**	**Total (n = 208)**	***P***
Age at transplantation, mean ± SD	62.5 ± 10.4	60.9 ± 13.4	61.6 ± 12.2	.357
Sex, n (%) Male Female	58 (63.7) 33 (36.3)	77 (65.8) 40 (34.2)	135 (64.9) 73 (35.1)	.756
BMI at transplantation, mean ± SD	26.4 ± 2.9	25.1 ± 4.3	25.8 ± 3.7	.175
Blood types, n (%) O A B AB	25 (29.1) 42 (46.2) 13 (14.3) 11 (12.1)	34 (29.1) 54 (46.2) 18 (15.4) 11 (9.4)	59 (28.4) 96 (46.2) 31 (14.9) 22 (10.6)	N/A
Smoking, n (%)	20 (22.0)	21 (17.9)	41 (19.8)	.775
Alcohol consumption, n (%*)*	12 (13.2)	9 (7.7)	21 (10.1)	.123
Observation duration since transplantation, months, mean ± SD	140 ± 75	109 ± 66	122 ± 72	**.002**
Observation duration since the diagnosis of neoplasm, months median (IQR)	26 (52)	27 (59)	26 (54)	.858
Primary disease,n (%) Alcohol HBV HBV/HDV coinfection HCV Fulminant hepatitis/drug Autoimmune hepatitis PSC PBC Wilson disease MASH Cryptogenic Other	10 (11.0) 38 (41.8) 10 (11.0) 9 (9.9) 1 (1.1) 3 (3.3) 2 (2.2) 1 (1.1) 2 (2.2) 2 (2.2) 11 (12.1) 2 (2.2)	10 (8.5) 45 (38.5) 8 (6.8) 13 (11.1) 1 (0.9) 6 (5.1) 2 (1.7) 3 (2.6) 0 (0) 9 (7.7) 12 (10.3) 8 (6.8)	20 (9.6) 83 (39.9) 18 (8.7) 22 (10.6) 2 (1.0) 9 (4.3) 4 (1.9) 4 (1.9) 2 (1.0) 11 (5.3) 23 (11.1) 10 (4.8)	N/A
HCC prior to transplantation, n (%)	23 (25.3)	36 (30.8)	59 (28.4)	.383
MELD score, mean ± SD	18.6 ± 6.2	17.7 ± 5.8	18.1 ± 5.9	.297
Comorbidities, n (%) DM HT CKD COPD CAD Other	14 (15.4) 15 (16.5) 2 (2.2) 0 (0) 2 (2.2) 6 (6.6)	21 (17.9) 13 (11.1) 1 (0.9) 3 (2.6) 1 (0.9) 5 (4.3)	35 (16.8) 28 (13.5) 3 (1.4) 3 (1.4) 3 (1.4) 11 (5.3)	.624 .260 .582 .258 .582 .539
Immunosuppressive medications, n (%) Tacrolimus MMF Everolimus Sirolimus Cyclosporin Azathioprine Prednisolone	76 (83.5) 47 (5.6) 27 (29.7) 0 (0) 7 (7.7) 2 (2.2) 23 (25.3)	96 (82.1) 49 (41.9) 42 (35.9) 2 (1.7) 10 (8.5) 1 (0.9) 20 (17.1)	172 (82.7) 96 (46.2) 69 (33.2) 2 (1.0) 17 (8.2) 3 (1.4) 43 (20.7)	.782 .161 .344 .505 .823 .582 .148
History of biopsy-proven rejection, n (%)	6 (6.6)	12 (10.3)	18 (8.7)	.458
Multiple de novo neoplasm, n (%)	4 (4.4)	3 (2.6)	7 (3.4)	.702
Neoplasm types, n (%) Hematologic Prostate Pancreas Dermatologic Lung GIT Bladder Gynecologic Head-neck Kaposi Other Multiple	19 (20.9) 5 (5.5) 3 (3.3) 20 (22.0) 10 (11.0) 5 (5.5) 3 (3.3) 5 (5.5) 3 (3.3) 2 (2.2) 12 (13.2) 4 (4.4)	23 (19.7) 5 (4.3) 4 (3.4) 28 (23.9) 11 (9.4) 6 (5.1) 2 (1.7) 8 (6.8) 6 (5.1) 1 (0.9) 20 (17.1) 3 (2.6)	42 (20.2) 10 (4.8) 7 (3.4) 48 (23.1) 21 (10.1) 11 (5.3) 5 (2.4) 13 (6.3) 9 (4.3) 3 (1.4) 32 (15.4) 7 (3.4)	N/A
Death, n (%)	31 (34.1)	42 (35.9)	73 (35.1)	.784

BCC, basal cell carcinoma; BMI, body mass index; CAD, coronary artery disease; CKD, chronic kidney disease; COPD, chronic obstructive pulmonary disease; DM, diabetes mellitus; GIT, gastrointestinal tract; HBV, hepatitis B virus; HCV, hepatitis C virus; HCC, hepatocellular carcinoma; HDV, hepatitis D virus; HT, hypertension; MASH, metabolic dysfunction-associated steatohepatitis; MELD, model for end-stage liver disease; MMF, mycophenolate mofetil; N/A, not applicable; IQR, interquartile range; PBC, primary biliary cholangitis; PSC, primary sclerosing cholangitis; SCC, squamous cell carcinoma.

**Table 2.** Neoplasm-Based Characteristics of De Novo Malignancies After Liver Transplantation According to Time of Onset (0-10 vs. >10 Years Post-transplant; 220 Neoplasms In 208 Patients)

	**Post-transplant 0-10 Years (n = 169)**	**Post-transplant >10 Years (n = 51)**	**Total (n = 220)**	***P***
Age at diagnosis, mean ± SD	57.0 ± 12.6	61.4 ± 10.5	58.0 ± 12.2	**.026**
Sex, n (%) Male Female	113 (66.9) 56 (33.1)	30 (58.8) 21 (41.2)	143 (65.0) 77 (35.0)	.291
BMI at diagnosis, mean ± SD	25.9 ± 4.5	26.6 ± 4.1	26.2 ± 4.3	.324
Donor type, n (%) Living Deceased	106 (62.7) 63 (37.3)	19 (37.3) 32 (62.7)	125 (56.8) 95 (43.2)	**.001**
Blood types, n (%) O A B AB	47 (27.8) 77 (45.6) 25 (14.8) 20 (11.8)	14 (27.5) 22 (43.1) 12 (23.5) 3 (5.9)	61 (27.7) 99 (45.0) 37 (16.8) 23 (10.5)	.368
Time elapsed since the transplantation months median (IQR)	48 (55)	168 (65)	63 (92)	**<.001**
Observation duration since the diagnosis of neoplasm months median (IQR)	29 (59)	28 (49)	29 (56)	.295
Primary disease,n (%) Alcohol HBV HBV/HDV coinfection HCV Fulminant hepatitis/drug Autoimmune hepatitis PSC PBC Wilson disease MASH Cryptogenic Other	17 (10.1) 64 (37.9) 12 (7.1) 18 (10.7) 1 (0.6) 7 (4.1) 3 (1.8) 4 (2.4) 2 (1.2) 11 (6.5) 22 (13.0) 8 (4.7)	5 (9.8) 22 (43.1) 7 (13.7) 4 (7.8) 1 (2.0) 2 (3.9) 1 (2.0) 0 (0) 0 (0) 1 (2.0) 6 (11.8) 2 (3.9)	22 (10.0) 86 (39.1) 19 (8.6) 22 (10.0) 2 (0.9) 9 (4.1) 4 (1.8) 4 (1.8) 2 (0.9) 12 (5.5) 28 (12.7) 10 (4.5)	N/A
HCC prior to transplantation, n (%)	49 (29.0)	11 (21.6)	60 (27.3)	.297
Comorbidities, n (%) DM HT CKD COPD CAD Other	29 (17.2) 21 (12.4) 2 (1.2) 1 (0.6) 2 (1.2) 10 (5.9)	8 (15.7) 9 (17.6) 1 (2.0) 2 (3.9) 1 (2.0) 1 (2.0)	37 (16.8) 30 (13.6) 3 (1.4) 3 (1.4) 3 (1.4) 11 (5.0)	.805 .341 .549 .135 .549 .464
Immunosuppressive medication, n (%) Tacrolimus MMF Everolimus Sirolimus Cyclosporin Azathioprine Prednisolone	144 (85.2) 80 (47.3) 53 (31.4) 2 (1.2) 12 (7.1) 1 (0.6) 37 (21.9)	38 (74.5) 23 (45.1) 18 (35.3) 0 (0) 6 (11.8) 2 (3.9) 6 (11.8)	182 (82.7) 103 (46.8) 71 (32.3) 2 (0.9) 18 (8.2) 3 (1.4) 43 (19.5)	.077 .779 .598 1.0 .380 .135 .110
History of biopsy-proven rejection, n (%)	13 (7.7)	5 (9.8)	18 (8.2)	.381
Multiple de novo neoplasm, n (%)	10 (5.9)	8 (15.7)	18 (8.2)	**.041**
Neoplasm groups, n (%) Skin Hematologic Non-skin solid	47 (27.8) 30 (17.8) 82 (55.4)	16 (31.4) 10 (19.6) 25 (49.0)	63 (28.6) 40 (18.2) 117 (53.2)	.622 .763 .873
Neoplasm types, n (%) Hematologic Prostate Pancreas BCC (skin) SCC (skin) Lung GIT Bladder Gynecologic Head-neck Kaposi Malignant melanoma Other	30 (17.8) 6 (3.6) 6 (3.6) 16 (9.5) 31 (18.3) 17 (10.1) 9 (5.3) 4 (2.4) 10 (5.9) 8 (4.7) 4 (2.4) 2 (1.2) 26 (15.4)	10 (19.6) 5 (9.8) 1 (2.0) 7 (13.7) 9 (17.6) 4 (7.8) 3 (5.9) 1 (2.0) 4 (7.8) 1 (2.0) 0 (0) 0 (0) 6 (11.8)	40 (18.2) 11 (5.0) 7 (3.2) 23 (10.5) 40 (18.2) 21 (9.5) 12 (5.5) 5 (2.3) 14 (6.4) 9 (4.1) 4 (1.8) 2 (0.9) 32 (14.5)	.763 .133 1.0 .384 .910 .789 1.0 1.0 .743 .688 .576 1.0 .520
Treatment approach, n (%) Surgery Chemotherapy Radiotherapy Combination Palliative	94 (55.6) 72 (42.6) 24 (14.2) 32 (18.9) 10 (5.9)	31 (60.8) 20 (39.2) 7 (13.7) 9 (17.6) 1 (2.0)	125 (56.8) 92 (41.8) 31 (14.1) 40 (18.2) 11 (5.0)	N/A
Death, n (%)	67 (39.6)	6 (11.8)	73 (33.2)	**<.001**

BCC, basal cell carcinoma; BMI, body-mass index; CAD, coronary artery disease; CKD, chronic kidney disease; COPD, chronic obstructive pulmonary disease; DM, diabetes mellitus; GIT, gastrointestinal tract; HBV, hepatitis B virus; HCV, hepatitis C virus; HCC, hepatocellular carcinoma; HDV, hepatitis D virus; HT, hypertension; MASH, metabolic dysfunction-associated steatohepatitis; MMF, mycophenolate mofetil; N/A, not applicable; IQR, interquartile range; PBC, primary biliary cholangitis; PSC, primary sclerosing cholangitis; SCC, squamous cell carcinoma.

**Table 3.** Neoplasm-based Subgroup Analysis Comparing De Novo Malignancies Diagnosed at ≤60 Vs. >60 Years of Age in Liver Transplant Recipients

	**Age ≤ 60 Years (n = 109)**	**Age > 60 Years (n = 111)**	***P***
Age, mean ± SD	53.8 ± 11.7	70.8 ± 5.3	**<.001**
Female sex, n (%)	47 (43.1)	30 (27.0)	**.012**
Donor type, n (%) Living Deceased	62 (56.9) 47 (43.1)	63 (56.8) 48 (43.2)	.985
Time elapsed since the transplantation, months median (IQR)	61 (94)	64 (81)	.083
Observation duration since the diagnosis of neoplasm, months median (IQR)	35 (62)	27 (52)	.914
HCC prior to transplantation, n (%)	18 (16.5)	42 (37.8)	**<.001**
Immunosuppressive medication n (%) Tacrolimus MMF Everolimus Sirolimus Cyclosporin Azathioprine Prednisolone	96 (88.1) 40 (36.7) 25 (22.9) 2 (1.8) 8 (7.3) 3 (2.8) 24 (22.0)	86 (77.5) 63 (56.8) 46 (41.4) 0 (0) 10 (9.0) 0 (0) 19 (17.1)	**.038** **.003** **.003** .244 .651 .120 .359
History of biopsy-proven rejection, n (%)	9 (8.3)	9 (8.1)	1.0
Neoplasm groups, n (%) Skin Hematologic Non-skin solid	25 (22.9) 26 (23.9) 58 (53.2)	38 (34.2) 14 (12.6) 59 (53.2)	.064 **.031** .893
Neoplasm types, n (%) Hematologic Prostate Pancreas BCC (skin) SCC (skin) Lung GIT Bladder Gynecologic Head-neck Kaposi Malign melanoma Other	26 (23.9) 1 (0.9) 5 (4.6) 8 (7.3) 16 (14.7) 10 (9.2) 6 (5.5) 2 (1.8) 7 (6.4) 5 (4.6) 3 (2.8) 1 (0.9) 19 (17.4)	14 (12.6) 10 (9.0) 2 (1.8) 15 (13.5) 24 (21.6) 11 (9.9) 6 (5.4) 3 (2.7) 7 (6.3) 4 (3.6) 1 (0.9) 1 (0.9) 13 (11.7)	**.031** **.010** .278 .135 .182 .853 .974 1.0 .972 .747 .367 1.0 .229
Treatment approach, n (%) Surgery Chemotherapy Radiotherapy Combination Palliative	51 (46.8) 49 (45.0) 13 (11.9) 15 (13.8) 5 (4.6)	74 (66.7) 43 (38.7) 18 (16.2) 25 (22.5) 6 (5.4)	**.003** .350 .361 .801 .757
Death, n (%)	43 (39.4)	30 (27.0)	.050

BCC, basal cell carcinoma; GIT, gastrointestinal tract; HCC, hepatocellular carcinoma; IQR, interquartile range; MMF, mycophenolate mofetil; SCC, squamous cell carcinoma.

**Table 4.** Neoplasm-based Subgroup Analysis of Clinical Characteristics, Management, and Outcomes of Skin Versus Non-skin De Novo Malignancies After Liver Transplantation

	**Skin Neoplasm (n = 63)**	**Non-skin Neoplasm (n = 157)**	***P***
Age, mean ± SD	60.0 ± 11.2	57.2 ± 12.6	.124
Sex, n (%) Male Female	39 (61.9) 24 (38.1)	104 (66.2) 53 (33.8)	.542
BMI, mean ± SD	26.4 ± 3.7	26.1 ± 4.7	.868
Donor type, n (%) Living Deceased	40 (63.5) 23 (36.5)	85 (54.1) 72 (45.9)	.206
Blood types, n (%) O A B AB	19 (30.2) 25 (39.7) 14 (22.2) 5 (7.9)	42 (26.8) 74 (47.1) 23 (14.6) 18 (11.5)	.422
Time elapsed since transplantation, months median (IQR)	72 (89)	61 (94)	.297
Observation duration since the diagnosis of neoplasm, months median (IQR)	45 (47)	22 (52)	**.001**
Primary disease,n (%) Alcohol HBV HBV/HDV coinfection HCV Fulminant hepatitis/drug Autoimmune hepatitis PSC PBC Wilson disease MASH Cryptogenic Other	5 (7.9) 24 (38.1) 7 (11.1) 6 (9.5) 2 (3.2) 2 (3.4) 0 (0) 0 (0) 0 (0) 6 (9.5) 8 (12.7) 2 (3.2)	17 (10.8) 62 (39.5) 12 (7.6) 16 (10.2) 0 (0) 6 (3.8) 4 (2.5) 4 (2.5) 2 (1.3) 6 (3.8) 20 (12.7) 8 (5.1)	N/A
HCC prior to transplantation, n (%)	15 (23.8)	45 (28.7)	.465
Comorbidities, n (%) DM HT CKD COPD CAD Other	11 (17.5) 7 (11.1) 1 (1.6) 1 (1.6) 1 (1.6) 2 (3.2)	26 (16.6) 23 (14.6) 2 (1.3) 2 (1.3) 2 (1.3) 9 (5.7)	.872 .489 1.0 1.0 1.0 .733
Immunosuppressive medication, n (%) Tacrolimus MMF Everolimus Sirolimus Cyclosporin Azathioprine Prednisolone	57 (90.5) 31 (49.2) 20 (31.7) 2 (3.2) 3 (4.8) 1 (1.6) 13 (20.6)	125 (79.6) 72 (45.9) 51 (32.5) 0 (0) 15 (9.6) 2 (1.3) 30 (19.1)	.054 .653 .916 .081 .241 1.0 .796
History of biopsy-proven rejection, n (%)	6 (9.5)	12 (7.6)	.890
Multiple de novo neoplasm, n (%)	12 (19.0)	6 (3.8)	**<.001**
Treatment approach, n (%) Surgery Chemotherapy Radiotherapy Combination Palliative	60 (95.2) 1 (1.6) 1 (1.6) 1 (1.6) 2 (3.2)	65 (41.4) 91 (58.0) 30 (19.1) 39 (24.8) 16 (10.2)	**<.001** **<.001** **.001** **.002** **.778**
Death, n (%)	5 (7.9)	68 (43.3)	**<.001**

BCC, basal cell carcinoma; BMI, body mass index; CAD, coronary artery disease; CKD, chronic kidney disease; COPD, chronic obstructive pulmonary disease; DM, diabetes mellitus; GIT, gastrointestinal tract; HBV, hepatitis B virus; HCV, hepatitis C virus; HCC, hepatocellular carcinoma; HDV, hepatitis D virus; HT, hypertension; MASH, metabolic dysfunction-associated steatohepatitis; MELD, model for end-stage liver disease; MMF, mycophenolate mofetil; N/A, not applicable; IQR, interquartile range; PBC, primary biliary cholangitis; PSC, primary sclerosing cholangitis; SCC, squamous cell carcinoma.

## Data Availability

The data that support the findings of this study are available on request from the corresponding author.
